# An integromic signature for lung cancer early detection

**DOI:** 10.18632/oncotarget.25227

**Published:** 2018-05-15

**Authors:** Qixin Leng, Yanli Lin, Min Zhan, Feng Jiang

**Affiliations:** ^1^ Department of Pathology, University of Maryland School of Medicine, Baltimore, MD 21201, USA; ^2^ Department of Epidemiology & Public Health, University of Maryland School of Medicine, Baltimore, MD 21201, USA

**Keywords:** diagnosis, early stage, lung cancer, plasma, biomarkers

## Abstract

We previously developed three microRNAs (miRs-21, 210, and 486-5p), two long noncoding RNAs (lncRNAs) (SNHG1 and RMRP), and two fucosyltransferase (FUT) genes (FUT8 and POFUT1) as potential plasma biomarkers for lung cancer. However, the diagnostic performance of the individual panels is not sufficient to be used in the clinics. Given the heterogeneity of lung tumors developed from multifactorial molecular aberrations, we determine whether integrating the different classes of molecular biomarkers can improve diagnosis of lung cancer. By using droplet digital PCR, we analyze expression of the seven genes in plasma of a development cohort of 64 lung cancer patients and 33 cancer-free individuals. The panels of three miRNAs (miRs-21, 210, and 486-5p), two lncRNAs (SNHG1 and RMRP), and two FUTs (FUT8 and POFUT1) have a sensitivity of 81-86% and a specificity of 84-87% for diagnosis of lung cancer. From the seven genes, an integromic plasma signature comprising miR-210, SNHG1, and FUT8 is developed that produces higher sensitivity (95.45%) and specificity (96.97%) compared with the individual biomarker panels (all p<0.05). The diagnostic value of the signature was confirmed in a validation cohort of 40 lung cancer patients and 29 controls, independent of stage and histological type of lung tumor, and patients’ age, sex, and smoking status (all p>0.05). The integration of the different categories of biomarkers might improve diagnosis of lung cancer.

## INTRODUCTION

Over 85% lung cancers are non-small cell lung cancers (NSCLC). NSCLC mainly consists of adenocarcinoma (AC) and squamous cell carcinoma (SCC). Tobacco smoking is the major cause of NSCLC. Since the prognosis for patients with lung cancer is strongly correlated to the tumor stage, diagnosing lung cancer at a curable stage can reduce the mortality [1]. The early detection of lung cancer in a large randomized trial using low-dose CT (LDCT) has revealed a 20% reduction in mortality as compared to chest X-rays [1]. However, LDCT is associated with over-diagnosis, excessive cost, and radiation exposure [2, 3]. The development of circulating biomarkers that can accurately and cost-effectively identify early stage lung cancer is required [4].

During tumor development, cancer cells undergo apoptosis and necrosis, and release tumor-associated molecules that can circulate in bloodstream. The tumors-derived molecules in plasma provide cell-free circulating cancer biomarkers. Regulatory non-coding RNAs (ncRNAs) can be classified into two major classes based on the transcript size: small ncRNAs (<200 bp) including microRNAs (miRNAs) and long ncRNAs (lncRNAs) (>200 bp) [5, 6]. Through different molecular mechanisms or pathways, the two types of ncRNAs have diverse and critical functions in tumorigenesis [7-10]. Furthermore, plasma miRNAs and lncRNAs directly released from primary lung tumors or the circulating lung cancer cells might provide cell-free biomarkers for lung cancer [8]. For instance, we recently developed a panel of three plasma miRNA biomarkers with 86% sensitivity and 87% specificity and a panel of two plasma lncRNA biomarkers with 83% sensitivity and 84% specificity for lung cancer early detection [7, 11-15].

Emerging evidences have demonstrated that aberrant glycosylation leads to cancer development and progression [16]. Fucosylation is the major type of glycosylation, and regulated by fucosyltransferases (FUTs) [16, 17]. We recently found that combined use of two plasma FUTs (FUT8 and POFUT1) had 81% sensitivity and 84% specificity for diagnosis of lung cancer, thus providing a new category of cell-free circulating biomarkers for lung cancer.

Since NSCLC is a heterogeneous disease and develops from multifactorial molecular aberrations [18], the analysis of one type of molecular changes may not achieve the performance required to move forward for clinical application. Indeed, although our individual panels of plasma biomarkers show promise for lung cancer diagnosis, their sensitivities (81-86%) and specificities (84-87%) are not sufficient to be used in the laboratory settings.

Because miRNAs, lncRNAs, and FUTs have highly diverse roles that drive the development of lung cancer [7-10, 16], we hypothesize that integrating the different classes of biomarkers may improve the early detection of lung cancer. Here we evaluate the individual and combined applications of the three categories of plasma molecular biomarkers for lung cancer.

## RESULTS

### The three individual panels of plasma biomarkers displayed a different level in NSCLC patients vs. smokers

Droplet Digital PCR (ddPCR) was used for quantification of the genes (miRs-21, 210, 486-5p, SNHG1, RMRP, FUT8, and POFUT1) in plasma of a development cohort of 64 lung cancer patients and 33 cancer-free individuals. All the seven genes generated at least 10,000 droplets in each well of the plasma samples. Therefore, the seven genes could be successfully ‘‘read’’ by ddPCR for their absolute quantification in plasma. These genes had a significantly different expression level in plasma of the NSCLC patients compared with the control individuals (all P<0.05). As a result, the individual genes resulted in 50.09 to 75.76% sensitivities and 63.64 to 90.91% specificities for detection of NSCLC (Table 1). Furthermore, the panel of three microRNA biomarkers (miRs-21, 210, and 486-5p) had an area under receiver operating characteristic curve (AUC) of 0.92 with 86.36% sensitivity and 87.88% specificity, the panel of two plasma lncRNA biomarkers (SNHG1 and RMRP) displayed 0.89 AUC with 83.33% sensitivity and 84.85% specificity, and the panel of two FUTs (FUT8 and POFUT1) exhibited an AUC of 0.85 with 81.82% sensitivity and 84.85% specificity for diagnosis of lung cancer (Table 2). The individual panels of the genes didn’t show special association with stage and histology of the NSCLC, age, gender, and smoking status of the participants (All p>0.05). The seven genes would be potential plasma biomarkers for lung cancer.

**Table 1:** Diagnostic performance of individual genes for lung cancer in a development cohort

	Sensitivity (95% CI)	Specificity (95% CI)
miRs-21	75.76% (63.64% to 85.46%)	63.64% (45.12% to 79.60%)
miR-210	50.09% (46.29% to 71.05%)	72.73% (54.48% to 86.70%)
miR-486-5p	72.73% (60.36% to 82.97%)	63.64% (45.12% to 79.60%)
SNHG1	75.76% (63.64% to 85.46%)	81.82% (64.54% to 93.02%)
RMRP	62.12% (49.34% to 73.78%)	90.91% (75.67% to 98.08%)
FUT8	71.21 (58.75% to 81.70%)	87.88% (71.80% to 96.60%)
POFUT1	60.61% (47.81% to 72.42%)	90.91% (75.67% to 98.08%)

Abbreviations: CI, confidence interval.

**Table 2:** The area under receiver operating characteristic curves (AUCs) of the individual panels of biomarkers and the plasma integromic signature in a development cohort

	AUC (95% CI)	Sensitivity (95% CI)	Specificity (95% CI)	Accuracy
A panel of 3 miRNAs	0.92 (0.87 to 0.97)	86.36% (75.69% to 93.57%)	87.88% (71.80% to 96.60%)	86.87% (78.59% to 92.82%)
A panel of 2 lncRNAs	0.89 (0.85 to 0.93)	83.33% (72.13% to 91.38%)	84.85% (68.10% to 94.89%)	83.84% (75.09% to 90.47%)
A panel of 2 FUTs	0.85 (0.78 to 0.91)	81.82% (70.39% to 90.24%)	84.85% (68.10% to 94.89%)	82.83% (73.94% to 89.67%)
An integromic signature	0.95 (0.91 to 0.99)	95.45% (87.29% to 99.05%)	96.97% (84.24% to 99.92%)	95.96% (89.98% to 98.89%)

Abbreviations: CI, confidence interval.

### An integromic plasma signature for lung cancer early detection

**Figure 1:**
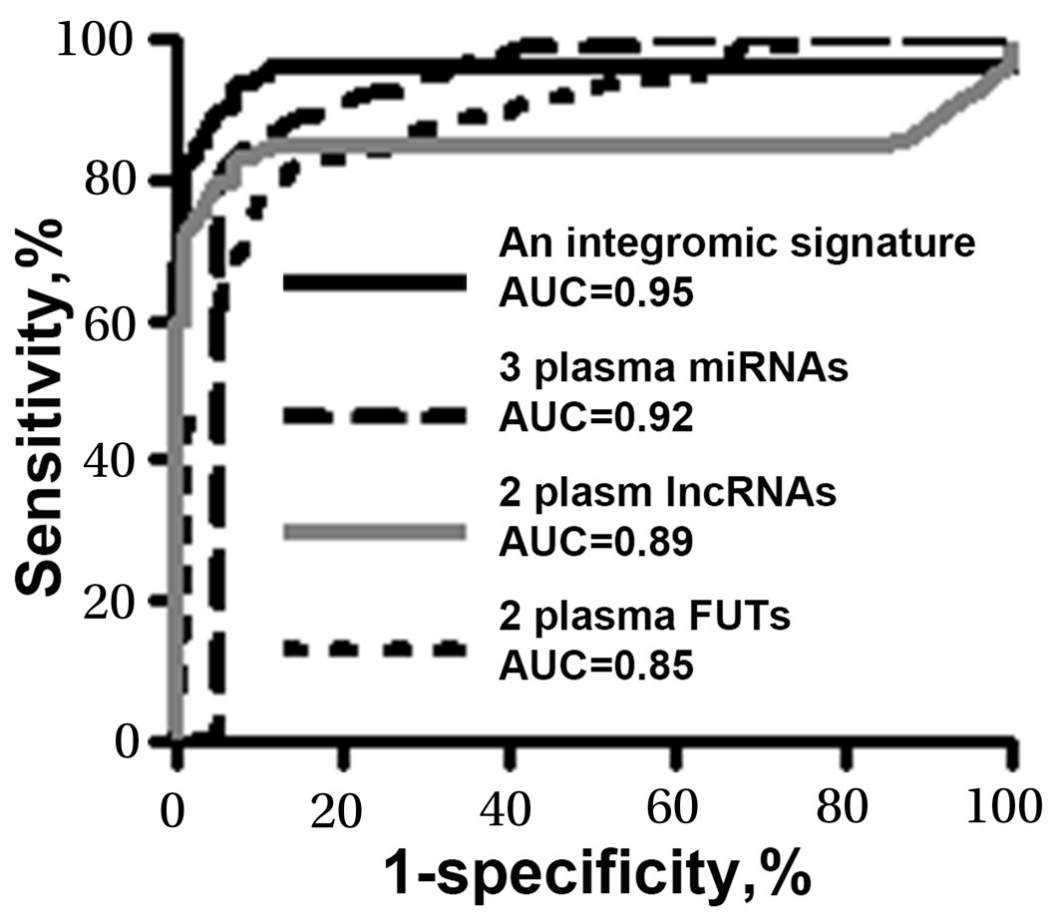
Diagnostic values of the individual panels of biomarkers and integromic plasma signature in a development cohort The integromic plasma biomarker signature yields a higher area under receiver operating characteristic curve (AUC) than does individual panels of biomarkers (All p<0.05).

We used logistic regression models with constrained parameters as in least absolute shrinkage and selection operator (LASSO) and AUCs to determine performance of different patterns of combining the genes. From the seven genes, one miRNA (miR-210), one lncRNA (SNHG1), and one FUT (FUT8) were selected as the best biomarkers (all P<0.001). A logisitic regression model with each of the different types of genes was developed as an integromic signature for diagnosing lung cancer: U=-7. 29+2.8^*^log (SNHG1) +3.83^*^log (FUT8) +3.36 ^*^log (miR-210). Combined analysis of the 3 biomarkers by using the logisitic regression model produced a higher AUC (0.97) (Figure 1) than did the individual panels of biomarkers (p<0.05). We used the highest Youden’s J index to set up corresponding cut-off value [19]. The optimal cut-off for the integromic signature was U=0.79. Any subject with U≥0.79 was classified as a lung cancer case. As a result, the integromic plasma signature yielded significantly higher sensitivity (95.45%), specificity (96.97%), and accuracy (95.96%) compared with the individual panels of biomarkers (all p<0.05) (Table 2). Furthermore, combined use of all the seven genes did not produce higher sensitivity and specificity compared with the integromic plasma signature (p>0.05). In addition, Pearson’s correlation analysis showed that the relationships among levels of the three genes were very low (All p>0.05), implying that the integration of the different classes of molecular biomarkers has complementary classification. Moreover, the integromic plasma signature had no special association with histological type of the NSCLC, age, gender, and smoking status of the participants (All p>0.05). The integromic signature did not show statistical difference of sensitivity and specificity for different stages of NSCLC (Supplementary Figure 1).

### Validating the integromic plasma signature for lung cancer detection

**Figure 2:**
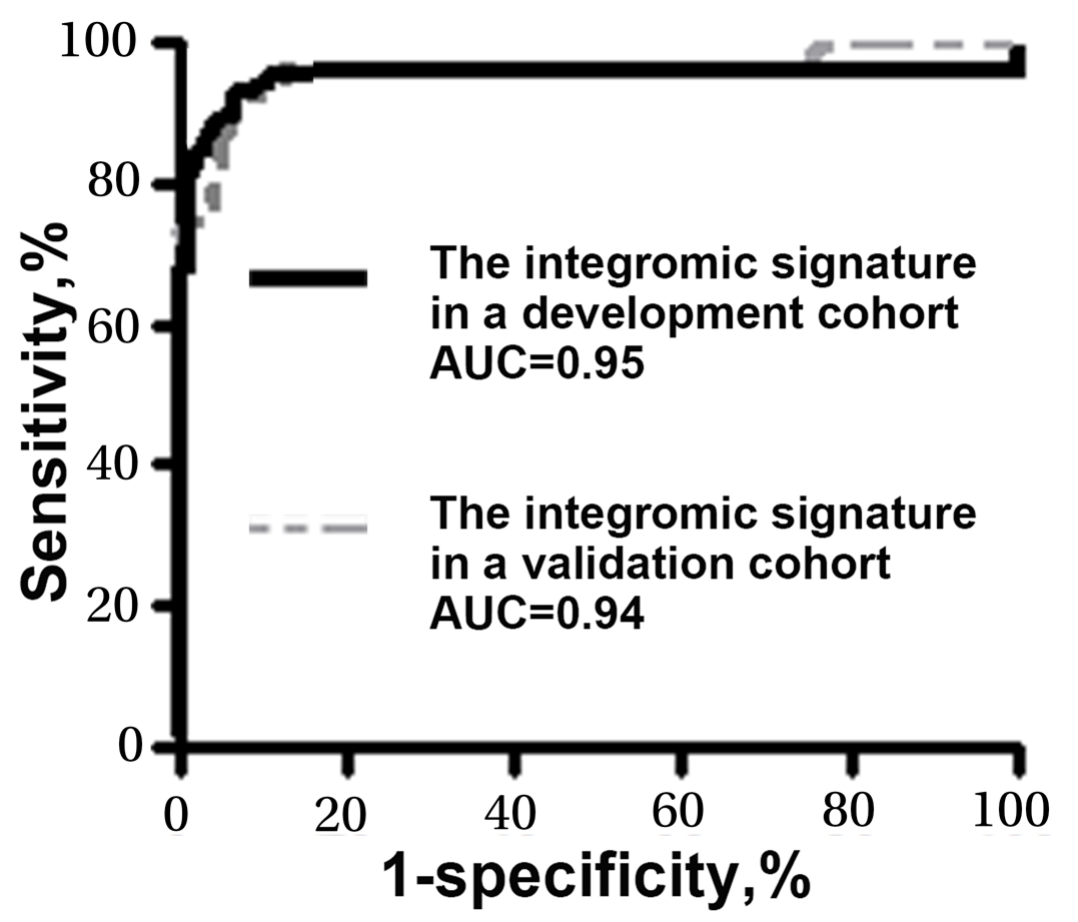
Comparison of AUCs of the integromic plasma signature for diagnosis of lung cancer in different cohorts The integromic plasma signature has no significant difference of AUCs in the development cohort (black line) vs. validation cohort (gray line) (0.95 vs. 0.94, p=0.46).

The plasma expression levels of the three genes (miR-210, SNHG1, and FUT8) were assessed by using ddPCR in a validation cohort of additional 40 NSCLC patients and 29 healthy controls. Combined analysis of the three genes by using the logisitic regression model created 0.94 AUC for lung cancer diagnosis. There was no significant difference between the develop cohort and validation cohort with regarding the signature’s AUCs (0.95 vs. 0.94, p=0.46) (Figure 2). In the validation cohort, the three genes used in combination could differentiate the NSCLC patients from healthy controls with a sensitivity of 95.00% (82.08% to 99.12%) and a specificity of 96.55% (80.37% to 99.82%). In line with the findings in the development cohort, the integromic plasma signature did not show statistical difference of sensitivity and specificity across different stages and subtypes of NSCLC (all p>0.05). Moreover, there was no association of expressions of the genes with the age, gender, or smoking status of the individuals (All p>0.05).

## DISCUSSION

Although showing promise, the use of the individual miRNA, lncRNA, or FUT biomarker panels alone has moderate sensitivities (81-86%) and specificities (84-87%). The miRNAs, lncRNAs, and FUTs have highly different functions in carcinogenesis [20-22]. Given the heterogeneous nature of lung cancer and the numerous cellular pathways involved, we hypothesize that integrating the different classes of molecular biomarkers may improve the early detection of lung cancer. Intriguingly, the integrated analysis of only one of each type of biomarkers by using a single platform (ddPCR) yields a significantly higher diagnostic performance compared with any panel of one type of genes. Furthermore, the correlations among the changes of the miRNAs, lncRNA, and FUT are very low, supporting that the diagnostic vales of the three classes of molecular alterations could be complementary to each other. Therefore, the observation confirms our hypothesis. Moreover, since the integromic plasma signature shows similar sensitivity and specificity in the early vs. advanced stages of NSCLC, it might be a useful approach for the early detection of lung cancer, a clinically challenging.

miR-210 stimulates a hypoxic phenotype and upsurges radioresistance in NSCLCs [21]. Hypoxia-induced miR-210 can regulate tumor cell susceptibility to cytolytic T-lymphocyte-mediated lysis by a mechanism involving its downstream targets PTPN1, HOXA1, and TP53I11 [23]. SNHG1 could promote NSCLC progression of lung cancer via miR-101-3p/SOX9/Wnt/β-catenin regulatory network and miR-145-5p/ MTDH axis [14, 22]. In addition, SNHG1 plays an oncogenic role in lung squamous cell carcinoma through ZEB1 signaling pathway by inhibiting TAp63 [24]. FUT8 inhibits the malignant behaviors of lung cancer cells and is involved in the regulation of dozens of genes associated with the malignancy through multiple mechanisms [20]. Upregulation of FUT8 could contribute to epithelial-mesenchymal transition via the transactivation of β-catenin/lymphoid enhancer-binding factor-1 (LEF-1) [20].

The study does have some limitations. 1), the sample size of the cohorts is small. We will perform a new study to prospectively validate the integromic signature for lung cancer early detection using a large population. 2), it is well known that lung cancer-associated molecular genetic changes are also related to chronic obstructive pulmonary disease (COPD) [25]. Many lung cancer patients who are smokers and cancer-free heavy smokers have COPD [25]. COPD could impact molecular genetic profiles in plasma of both lung cancer patients and cancer-free heavy smokers. In this present project, there is no COPD information of the cases and controls enrolled. Therefore, we are not able to evaluate if the biomarkers identified in the study are associated with COPD. We will recruit lung cancer patients and cancer-free smokers who have COPD, and determine if COPD is the confounding effect on the molecular changes. 3), the early detection of NSCLC using LDCT followed by appropriate treatments can significantly reduce lung cancer mortality in smokers [1]. LDCT is now recommended for lung cancer screening in smokers. Yet LDCT has a low specificity for the early detection of lung cancer, presenting a major clinical challenge [1]. The participants enrolled in this project are not representative of the smokers in LDCT screening setting for lung cancer. We will perform a prospective trial to determine if the integromic signature could improve the spesitivity of LDCT for the early deteciton of lung cancer in smokers.

## MATERIALS AND METHODS

### Patients and clinical specimens

Using a protocol approved by the local Institutional Review Boards Institutional Review Boards, we recruited lung cancer patients and cancer-free smokers according to the inclusion and/or exclusion criteria recommended by U.S. Preventive Services Task Force [26]. Briefly, we enrolled smokers between the ages of 55-80 who had at least a 30 pack-year smoking history and were former smokers (quit within 15 years). Exclusion criteria included pregnancy, current pulmonary infection, surgery within 6 months, radiotherapy within 1 year, and life expectancy of < 1 year. We collected blood in BD Vacutainer spray-coated K2EDTA Tubes (BD, Franklin Lakes, NJ) and prepared plasma using the standard operating protocols developed by The NCI-Early Detection Research Network [27]. The specimens were processed within 2 hours of collection by centrifugation at 1,300 X g for 10 minutes at 4°C. The surgical-pathologic staging of NSCLC was used as the ground truth according to the TNM classification of the International Union Against Cancer (UICC) with the American Joint Committee on Cancer (AJCC) and the International Staging System for Lung Cancer [28, 29]. A total of 106 NSCLC patients and 62 cancer-free smokers were recruited. Among the cancer patients, 27 patients were female and 79 were male. Twenty-four had stage I NSCLC, 19 with stage II, 28 with stage III, 30 with stage IV, and 5 with unknown stage. Fifty-six lung cancer patients were diagnosed with AC, while 40 with SCC. Of the cancer-free smokers, 16 patients were female and 46 were male. There were no significant differences of age, gender and smoking status between the NSCLC patients and cancer-free smokers. The cases and controls were randomly grouped into two cohorts: a development cohort and a validation cohort. The development cohort consisted of 66 lung cancer patients and 33 cancer-free smokers, while the validation cohort comprised 40 lung cancer patients and 29 cancer-free smokers. The demographic and clinical variables of the two cohorts are shown in Table 3.

**Table 3:** Characteristics of a development cohort and a validation cohort

	A development cohort		
	NSCLC cases (n = 66)	Controls (n = 33)	P-value
Age	67.68 (SD 9.23)	62.70 (SD 15.33)	0.16
Sex			0.39
Female	17	8	
Male	49	25	
Smoking pack-years (median)	33.6	32.69	0.17
Stage			
Stage I	15		
Stage II	11		
Stage III	17		
Stage IV	19		
Unknown	4		
Histological type			
Adenocarcinoma	34		
Squamous cell carcinoma	32		

	A validation cohort		
	NSCLC cases (n = 40)	Controls (n = 29)	P-value
Age	64.57 (SD 9.28)	63.76 (SD 13.49)	0.28
Sex			0.46
Female	10	8	
Male	30	21	
Smoking pack-years (median)	33.68	31.63	0.23
Stage			
Stage I	9		
Stage II	8		
Stage III	11		
Stage IV	11		
Unknown	1		
Histological type			
Adenocarcinoma	22		
Squamous cell carcinoma	18		

Abbreviations: NSCLC, non-small cell lung cancer.

### ddPCR

RNA was extracted from plasma by using Trizol LS reagent (Invitrogen Carlsbad, CA) and RNeasy Mini Kit (Qiagen, Hilden, Germany) [11, 12]. The qualification and quantification of RNA were assessed by using Biospectrometer (Hutchinson Technology Inc, Hutchinson, MN) and Electrophoresis Bioanalyzer (Agilent Technologies, Foster City, CA). Reverse Transcriptase (RT) was carried out to generate cDNA by using a RT Kit (Applied Biosystems, Foster City, CA) [11, 12]. ddPCR for analysis of expression level of the genes was performed as described in our published works by using a QX200™ Droplet Digital™ PCR System (Bio-Rad, Hercules, CA) [11-15, 30-50]. Briefly, PCR reaction mix containing cDNA was partitioned into aqueous droplets in oil via the QX100 Droplet Generator, and then transferred to a 96-well PCR plate. A two-step thermocycling protocol (95°C ×10min; 40 cycles of [94°C ×30s, 60°C ×60s], 98°C ×10 min) was undertaken in a Bio-Rad C1000 (Bio-Rad, Pleasanton, CA). The PCR plate was then transferred to the QX100 Droplet Reader for automatic reading of samples in all wells. Copy number of each gene per μl PCR reaction was directly determined. Primers and probes of the targeted genes are shown in Supplementary Table 1. We used QuantaSoft 1.7.4 analysis software (Bio-Rad) and Poisson statistics to compute droplet concentrations (copies/μL). Only genes that had at least 10,000 droplets were considered to be robustly detectable by ddPCR in plasma and subsequently underwent further analysis [31]. All assays were done in triplicates, and one no-template control and two interplate controls were carried along in each experiment.

### Statistical analysis

To estimate sample size, we set AUC of H0 (the null hypothesis) at 0.5. H1 represented the alternative hypothesis. To have a high reproducibility with adequate precision, we required ≥28 subjects per group. With this sample size, we would have 85% power to detect an AUC of 0.75 at the 2% significance level. Therefore, the sample size in the two cohorts could have enough statistical power. Pearson’s correlation analysis was applied to assess relationship between gene expressions and demographic and clinical characteristics of the patients and control individuals. AUCs were used to determine accuracy, sensitivity, and specificity of each gene. We used the highest Youden’s J index (sum of sensitivity and specificity—1) to set up corresponding cut-off value [19]. Logistic regression models with constrained parameters as in LASSO were used to eliminate the irrelevant genes, develop composite panels of biomarkers, and optimize a signature with the highest sensitivity and specificity. To compare the signature and our previously developed plasma biomarker panels, we compared their AUCs to determine the sensitivity and specificity as previously described [15].

## CONCLUSIONS

Given the heterogeneous nature of NSCLC developed from multifactorial molecular aberrations, we have for the first time demonstrated that the integration of miRNA, lncRNA, and FUT biomarkers could provide an efficient approach for diagnosis of lung cancer. Nonetheless, a large multi-center clinical project to prospectively validate the full utility of the integromic signature is required.

## SUPPLEMENTARY MATERIALS FIGURE AND TABLE



## References

[1] Aberle DR, Adams AM, Berg CD, Black WC, Clapp JD, Fagerstrom RM, Gareen IF, Gatsonis C, Marcus PM, Sicks JD (2011). Reduced lung-cancer mortality with low-dose computed tomographic screening. N Engl J Med.

[2] Patz EF, Pinsky P, Gatsonis C, Sicks JD, Kramer BS, Tammemagi MC, Chiles C, Black WC, Aberle DR (2014). Overdiagnosis in low-dose computed tomography screening for lung cancer. JAMA Intern Med.

[3] Aberle DR, Berg CD, Black WC, Church TR, Fagerstrom RM, Galen B, Gareen IF, Gatsonis C, Goldin J, Gohagan JK, Hillman B, Jaffe C, Kramer BS (2011). The National Lung Screening Trial: overview and study design. Radiology.

[4] Hubers AJ, Prinsen CF, Sozzi G, Witte BI, Thunnissen E (2013). Molecular sputum analysis for the diagnosis of lung cancer. Br J Cancer.

[5] Carthew RW, Sontheimer EJ (2009). Origins and Mechanisms of miRNAs and siRNAs. Cell.

[6] Orom UA, Shiekhattar R (2013). Long noncoding RNAs usher in a new era in the biology of enhancers. Cell.

[7] Shen J, Jiang F (2012). Applications of MicroRNAs in the Diagnosis and Prognosis of Lung Cancer. Expert Opin Med Diagn.

[8] Mitchell PS, Parkin RK, Kroh EM, Fritz BR, Wyman SK, Pogosova-Agadjanyan EL, Peterson A, Noteboom J, O'Briant KC, Allen A, Lin DW, Urban N, Drescher CW (2008). Circulating microRNAs as stable blood-based markers for cancer detection. Proc Natl Acad Sci U S A.

[9] Huarte M (2015). The emerging role of lncRNAs in cancer. Nat Med.

[10] Yanaihara N, Caplen N, Bowman E, Seike M, Kumamoto K, Yi M, Stephens RM, Okamoto A, Yokota J, Tanaka T, Calin GA, Liu CG, Croce CM (2006). Unique microRNA molecular profiles in lung cancer diagnosis and prognosis. Cancer Cell.

[11] Shen J, Todd NW, Zhang H, Yu L, Lingxiao X, Mei Y, Guarnera M, Liao J, Chou A, Lu CL, Jiang Z, Fang H, Katz RL, Jiang F (2011). Plasma microRNAs as potential biomarkers for non-small-cell lung cancer. Lab Invest.

[12] Shen J, Liu Z, Todd NW, Zhang H, Liao J, Yu L, Guarnera MA, Li R, Cai L, Zhan M, Jiang F (2011). Diagnosis of lung cancer in individuals with solitary pulmonary nodules by plasma microRNA biomarkers. BMC Cancer.

[13] Shen J, Liao J, Guarnera MA, Fang H, Cai L, Stass SA, Jiang F (2014). Analysis of MicroRNAs in sputum to improve computed tomography for lung cancer diagnosis. J Thorac Oncol.

[14] Leng Q, Lin Y, Jiang F, Lee CJ, Zhan M, Fang H, Wang Y (2017). A plasma miRNA signature for lung cancer early detection. Oncotarget.

[15] Lin Y, Leng Q, Jiang Z, Guarnera MA, Zhou Y, Chen X, Wang H, Zhou W, Cai L, Fang H, Li J, Jin H, Wang L (2017). A classifier integrating plasma biomarkers and radiological characteristics for distinguishing malignant from benign pulmonary nodules. Int J Cancer.

[16] Pinho SS, Reis CA (2015). Glycosylation in cancer: mechanisms and clinical implications. Nat Rev Cancer.

[17] Kirwan A, Utratna M, O'Dwyer ME, Joshi L, Kilcoyne M (2015). Glycosylation-Based Serum Biomarkers for Cancer Diagnostics and Prognostics. Biomed Res Int.

[18] Kadara H, Wistuba II (2012). Field cancerization in non-small cell lung cancer: implications in disease pathogenesis. Proc Am Thorac Soc.

[19] Youden WJ (1950). Index for rating diagnostic tests. Cancer.

[20] Chen CY, Jan YH, Juan YH, Yang CJ, Huang MS, Yu CJ, Yang PC, Hsiao M, Hsu TL, Wong CH (2013). Fucosyltransferase 8 as a functional regulator of nonsmall cell lung cancer. Proc Natl Acad Sci U S A.

[21] Grosso S, Doyen J, Parks SK, Bertero T, Paye A, Cardinaud B, Gounon P, Lacas-Gervais S, Noel A, Pouyssegur J, Barbry P, Mazure NM, Mari B (2013). MiR-210 promotes a hypoxic phenotype and increases radioresistance in human lung cancer cell lines. Cell Death Dis.

[22] Cui Y, Zhang F, Zhu C, Geng L, Tian T, Liu H (2017). Upregulated lncRNA SNHG1 contributes to progression of non-small cell lung cancer through inhibition of miR-101-3p and activation of Wnt/beta-catenin signaling pathway. Oncotarget.

[23] Noman MZ, Buart S, Romero P, Ketari S, Janji B, Mari B, Mami-Chouaib F, Chouaib S (2012). Hypoxia-inducible miR-210 regulates the susceptibility of tumor cells to lysis by cytotoxic T cells. Cancer Res.

[24] Zhang HY, Yang W, Zheng FS, Wang YB, Lu JB (2017). Long non-coding RNA SNHG1 regulates zinc finger E-box binding homeobox 1 expression by interacting with TAp63 and promotes cell metastasis and invasion in Lung squamous cell carcinoma. Biomed Pharmacother.

[25] Carr LL, Jacobson S, Lynch DA, Foreman MG, Flenaugh EL, Hersh CP, Sciurba FC, Wilson DO, Sieren JC, Mulhall P, Kim V, Kinsey CM, Bowler RP (2018). Features of COPD as Predictors of Lung Cancer. Chest.

[26] Humphrey LL, Deffebach M, Pappas M, Baumann C, Artis K, Mitchell JP, Zakher B, Fu R, Slatore CG (2013). Screening for lung cancer with low-dose computed tomography: a systematic review to update the US Preventive services task force recommendation. Ann Intern Med.

[27] Tuck MK, Chan DW, Chia D, Godwin AK, Grizzle WE, Krueger KE, Rom W, Sanda M, Sorbara L, Stass S, Wang W, Brenner DE (2009). Standard operating procedures for serum and plasma collection: early detection research network consensus statement standard operating procedure integration working group. J Proteome Res.

[28] Leong SS, Rocha Lima CM, Sherman CA, Green MR (1999). The 1997 International Staging System for non-small cell lung cancer: have all the issues been addressed?. Chest.

[29] Bulzebruck H, Bopp R, Drings P, Bauer E, Krysa S, Probst G, van Kaick G, Muller KM, Vogt-Moykopf I (1992). New aspects in the staging of lung cancer. Prospective validation of the International Union Against Cancer TNM classification. Cancer.

[30] Li N, Ma J, Guarnera MA, Fang H, Cai L, Jiang F (2014). Digital PCR quantification of miRNAs in sputum for diagnosis of lung cancer. J Cancer Res Clin Oncol.

[31] Ma J, Li N, Guarnera M, Jiang F (2013). Quantification of Plasma miRNAs by Digital PCR for Cancer Diagnosis. Biomark Insights.

[32] Su J, Leng Q, Lin Y, Ma J, Jiang F, Lee CJ, Fang H (2018). Integrating Circulating Immunological and Sputum Biomarkers for the Early Detection of Lung Cancer. Biomark Cancer.

[33] Su Y, Fang H, Jiang F (2016). Integrating DNA methylation and microRNA biomarkers in sputum for lung cancer detection. Clin Epigenetic.

[34] Su Y, Guarnera MA, Fang H, Jiang F (2016). Small non-coding RNA biomarkers in sputum for lung cancer diagnosis. Clin Epigenetics.

[35] Su J, Anjuman N, Guarnera MA, Zhang H, Stass SA, Jiang F (2015). Analysis of Lung Flute-collected Sputum for Lung Cancer Diagnosis. Biomark Insights.

[36] Su J, Liao J, Gao L, Shen J, Guarnera MA, Zhan M, Fang H, Stass SA, Jiang F (2016). Analysis of small nucleolar RNAs in sputum for lung cancer diagnosis. Oncotarget.

[37] Xing L, Su J, Guarnera MA, Zhang H, Cai L, Zhou R, Stass SA, Jiang F (2015). Sputum microRNA biomarkers for identifying lung cancer in indeterminate solitary pulmonary nodules. Clin Cancer Res.

[38] Pine PS, Lund SP, Stass SA, Kukuruga D, Jiang F, Sorbara L, Srivastava S, Salit M (2018). Cell-based reference samples designed with specific differences in microRNA biomarkers. BMC Biotechnol.

[39] Anjuman N, Li N, Guarnera M, Stass SA, Jiang F (2013). Evaluation of lung flute in sputum samples for molecular analysis of lung cancer. Clin Transl Med.

[40] Yu L, Todd NW, Xing L, Xie Y, Zhang H, Liu Z, Fang H, Zhang J, Katz RL, Jiang F (2010). Early detection of lung adenocarcinoma in sputum by a panel of microRNA markers. Int J Cancer.

[41] Jiang F, Todd NW, Li R, Zhang H, Fang H, Stass SA (2010). A panel of sputum-based genomic marker for early detection of lung cancer. Cancer Prev Res (Phila).

[42] Xing L, Todd NW, Yu L, Fang H, Jiang F (2010). Early detection of squamous cell lung cancer in sputum by a panel of microRNA markers. Mod Pathol.

[43] Xie Y, Todd NW, Liu Z, Zhan M, Fang H, Peng H, Alattar M, Deepak J, Stass SA, Jiang F (2010). Altered miRNA expression in sputum for diagnosis of non-small cell lung cancer. Lung Cancer.

[44] Jiang F, Todd NW, Qiu Q, Liu Z, Katz RL, Stass SA (2009). Combined genetic analysis of sputum and computed tomography for noninvasive diagnosis of non-small-cell lung cancer. Lung Cancer.

[45] Li H, Jiang Z, Leng Q, Bai F, Wang J, Ding X, Li Y, Zhang X, Fang H, Yfantis HG, Xing L, Jiang F (2017). A prediction model for distinguishing lung squamous cell carcinoma from adenocarcinoma. Oncotarget.

[46] Ma J, Guarnera MA, Zhou W, Fang H, Jiang F (2017). A Prediction Model Based on Biomarkers and Clinical Characteristics for Detection of Lung Cancer in Pulmonary Nodules. Transl Oncol.

[47] Li R, Todd NW, Qiu Q, Fan T, Zhao RY, Rodgers WH, Fang HB, Katz RL, Stass SA, Jiang F (2007). Genetic deletions in sputum as diagnostic markers for early detection of stage I non-small cell lung cancer. Clin Cancer Res.

[48] Ma J, Lin Y, Zhan M, Mann DL, Stass SA, Jiang F (2015). Differential miRNA expressions in peripheral blood mononuclear cells for diagnosis of lung cancer. Lab Invest.

[49] Gao L, Ma J, Mannoor K, Guarnera MA, Shetty A, Zhan M, Xing L, Stass SA, Jiang F (2015). Genome-wide small nucleolar RNA expression analysis of lung cancer by next-generation deep sequencing. Int J Cancer.

[50] Ma J, Mannoor K, Gao L, Tan A, Guarnera MA, Zhan M, Shetty A, Stass SA, Xing L, Jiang F (2014). Characterization of microRNA transcriptome in lung cancer by next-generation deep sequencing. Mol Oncol.

