# Supplementary material for: An integromic signature for lung cancer early detection
Source: Oncotarget. 2018 May 15;9(37):24684–92. doi: 10.18632/oncotarget.25227 (PMC5973873; doi:10.18632/oncotarget.25227)
Supplement: Supplementary file 1 [file oncotarget-09-24684-s001.pdf]

An integromic signature for lung cancer early detection

SUPPLEMENTARY MATERIALS

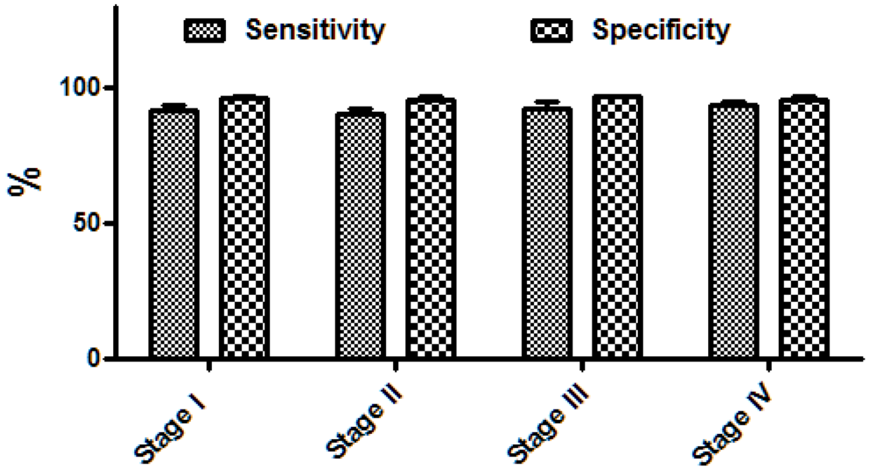

Supplementary Figure 1: Diagnostic performance of the integromic biomarker signature for different stages of lung cancer in a development cohort. The integromic signature does not show statistical difference of sensitivity and specificity among different stages of lung cancer. Error bars represent 95% confidence intervals. All p>0.05.

Supplementary Table 1: Primer sequences of the seven targeted genes

Genes	Forward	Reverse
mir-21	5'-TAGCTTATCAGACTGATGTTGA-3'	mRQ 3' primer
mir-210	5'-CTGTGCGTGTGACAGCGGCTGA-3'	mRQ 3' primer
mir-486-5p	5'-TCCTGTACTGAGCTGCCCCGAG-3'	mRQ 3' primer
RMRP	5'-TGCATACGCACGTAGACATT-3'	5'-TGCATACGCACGTAGACATT-3'
FUT8	5'-GTCAGGTGAAGTGAAGGACAA-3'	5'-CTGGTACAGCCAAGGGTAAAT-3'
SNHG1	5'-CCTTCAGAGCTGAGAGGTACTA-3'	5'-CTCAAACTCCTCTTGGGCTTTA-3'
POFUT1	5'-CAGCGAAGCCCAGATAAGAA-3'	5'-CTGTAGGAAGCACTGAAGGAAA-3'
